# Electrochemical Detection for Isothermal Loop-Mediated Amplification of Pneumolysin Gene of *Streptococcus pneumoniae* Based on the Oxidation of Phenol Red Indicator

**DOI:** 10.1021/acs.analchem.2c02127

**Published:** 2022-09-15

**Authors:** Andrea González-López, María Dolores Cima-Cabal, Pablo Rioboó-Legaspi, Estefanía Costa-Rama, María del Mar García-Suárez, M. Teresa Fernández-Abedul

**Affiliations:** † Departamento de Química Física y Analítica, Universidad de Oviedo, Avda. Julián Clavería 8, Oviedo33006, Spain; ‡ Escuela Superior de Ingeniería y Tecnología, Universidad Internacional de La Rioja, Avda. de La Paz 137, Logroño26006, Spain

## Abstract

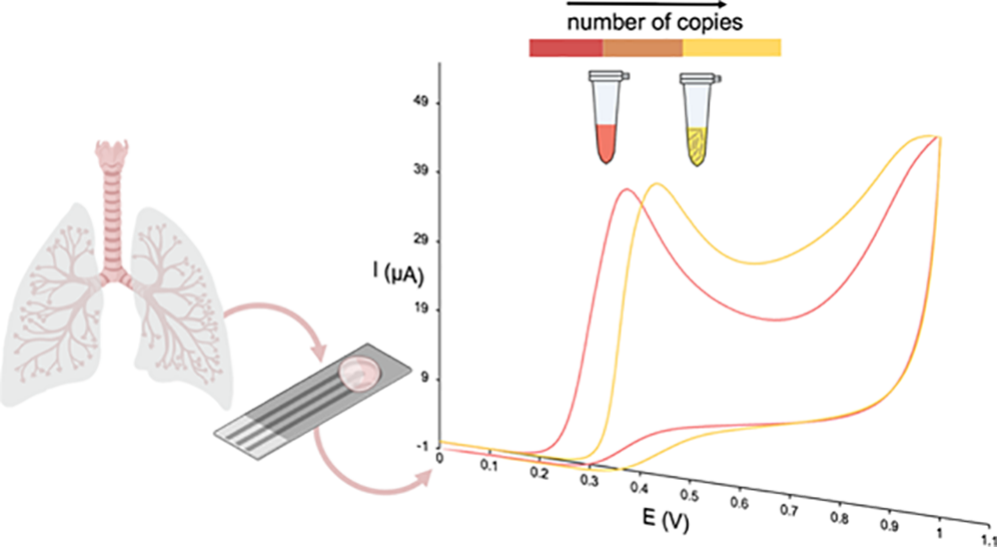

A highly sensitive electrochemical methodology for end-point detection of loop-mediated isothermal nucleic acid amplification reactions was developed. It is based on the oxidation process of phenol red (PR), commonly used as a visual indicator. The dependence of its redox process on pH, which changes during amplification, allows performing quantitative measurements. Thus, the change in the oxidation potential of PR during the amplification is used, for the first time, as the analytical signal that correlates with the number of initial DNA copies. As a proof-of-concept, the amplification of the pneumolysin gene from *Streptococcus pneumoniae*, one of the main pathogens causing community-acquired pneumonia, is performed. Combination of isothermal amplification with electrochemical detection, performed on small-size flexible electrodes, allows easy decentralization. Adaptation to the detection of other pathogens causing infectious diseases would be very useful in the prevention of future epidemics.

One of the things that we have learnt from this COVID-19 pandemic situation is that access to accurate, rapid, affordable, and decentralized diagnostic tests for community screenings is a mandatory requirement in the fight against infectious diseases.^1^ Current and future biological threats causing infectious diseases require being as prepared as possible, that meaning diagnosing them fast to avoid pathogen dissemination and to seek for prompt recovery. Point-of-care (POC) technology fits perfectly for this purpose since it offers unique advantages such as low analysis time and simple and cost-effective fabrication of devices.^2,3^ Diagnostic tests are commonly based on molecular biology techniques, with PCR (polymerase chain reaction) considered as the gold standard,^4−6^ and also assays for rapid (although less sensitive) detection of antigens. Although the possibility of increasing enormously the sensitivity of the assays by copying exponentially the genetic material has spread the PCR methodology, the need to use thermocyclers with exhaustive temperature controls hampers the idea of POC tests for decentralized detection. To overcome this problem, alternative nucleic acid isothermal amplification techniques have been developed. Maintaining a constant temperature simplifies the equipment, not being necessary to reach different values. This avoids dead times, and energy requirements are much lower, a relevant issue in the context of sustainable analysis.

In the loop-mediated isothermal amplification (LAMP) strategy, DNA is amplified at a fixed temperature through the repetition of two types of elongation reactions occurring at the terminal loop region and the binding and elongation of new primers.^7,8^ Indicator molecules such as colored or fluorescent dyes, allowing visual discrimination between positive and negative amplifications, are usually added to the amplification reaction.^9^ Its simplicity has spread a wide range of applications not only for clinical POC devices (with special interest in those applicable to low-resource areas) but also for on-site food safety and environmental monitoring.^10,11^

Applications to the diagnosis of infectious diseases, either those caused by bacteria or viruses, are being developed. This is the case of the detection of *Legionella*,^12^*Mycoplasma pneumoniae*,^13^ or *Staphylococcus aureus*, *Escherichia coli*, *Klebsiella pneumoniae* and *Pseudomonas aeruginosa*(14) in respiratory samples. The LAMP methodology has shown, in all cases, promising results. As regard viruses, LAMP or reverse transcriptase-LAMP procedures are employed depending on whether the genetic material is DNA or RNA. The most obvious example is the case of SARS-CoV-2,^15−17^ but also zika,^18^ influenza,^19^ or African swine fever^20^ viruses have been detected with this methodology. In all cases, the LAMP technique was clearly presented as a reliable, cheaper, and faster alternative to conventional diagnostic methods.

Detection of LAMP reactions using different principles, mainly optical, either by the naked eye or spectrophotometrically, has been reported using, for example, hydroxynaphtol blue or phenol red (PR) indicators that produce a color change.^9^ However, to achieve real decentralization, easy-to-miniaturize equipment with integration of amplification and detection in the same device is required. Electrochemical detection (ED) fits perfectly with these goals. Portable sensors based on electrochemical measurements performed with printed electrodes and small measuring devices are very useful in the field of diagnosis of infectious diseases.^21^ There are examples in which the product of the LAMP reaction is deposited on an electrochemical cell after interaction with an electroactive indicator (e.g., H33258 dye^10,22^). Capturing amplicons with methylene blue (MB) embedded on an appropriately modified electrode produces a strong electrochemical signal.^23^ Alternatively, since MB does not interfere with LAMP, it could be previously added in the master mix.^24,25^ Introducing a miniaturized cell in the solution after the LAMP reaction^26^ or depositing the product of the reaction on a cell with screen-printed electrodes^25^ allows measuring its redox process. Differential diffusion behavior of free and bound indicator molecules is the basis of the detection. This also happens when other types of indicators such as metallic complexes (e.g., osmium^27^) are employed. Enzymes (e.g., horseradish peroxidase) can also be incorporated in the amplicons,^28^ allowing the measurement of a product of an enzymatic reaction, proportional to the concentration of copies.

A simpler and different approach deals with the detection of ions that are released during the amplification. This is the case of pyrophosphate ions^29,30^ and protons. The use of DNA structures which undergo conformational changes with pH is one strategy that is based on the employment of complementary DNA strands labelled with ferrocene as the indicator molecule.^31,32^ Electroactive polymers that are pH-sensitive have also been employed.^33^ Direct measurement of pH using proton-selective graphene-modified screen-printed electrodes^34^ or metal-oxide field-effect transistors^35^ has been also reported.

In this context, PR is a pH indicator dye used for the detection of nucleic acid amplification in LAMP reactions.^9^ Strong strand-displacing DNA polymerases (like Bst DNA/RNA polymerase) incorporate a deoxynucleoside triphosphate into the nascent DNA releasing a pyrophosphate moiety and a hydrogen ion. In a weakly buffered solution, the pH decreases, due to the release of protons, from an initial value of approximately 8.0 to a final pH of 6.0–6.5, depending on the number of copies generated.^9^ Therefore, PR is helpful for visual detection of the amplification since it has discernible color transition in the neutral range, changing from red to yellow. Besides its properties as a colorimetric indicator, PR is also an electroactive species that shows a useful redox process,^36−38^ and therefore dual detection (visual and instrumental) is possible.

In this work, we report the ED of PR at end-point LAMP reactions of the pneumolysin gene (ply) of *Streptococcus pneumoniae*, using a novel strategy. Although some works have already combined LAMP reaction with ED, this is the first time that PR, used as the indicator for naked-eye LAMP detection, is used also for ED.

The ply gene codifies one main virulence factor of this bacteria. Pneumolysin is a 53 KDa protein with 471 amino acids and 4 domains which is present in all pneumococcal strains. Its genetic sequence is quite stable, which converts it in an ideal molecule as a diagnosis target. Ply belongs to the cholesterol-dependent citolysin family, a protein group that attacks cells with cholesterol in their membranes forming pores of 350–450 Å diameter, causing cell damage and death.^39^

Community-acquired pneumonia (CAP) is a low respiratory tract infection, considered as one of the six main causes of death in high-income countries. Around 10% of CAP patients that are hospitalized will require intensive care management. *S. pneumoniae* is a leading cause of death from pneumonia in both adults and children.^40^ Since the introduction of pneumococcal conjugate vaccines, the mortality rate has dropped. However, prevalence still remains high in children <5 years of age and adults over 65, the population with higher risk of severe illness.^41^ The recent SARS-CoV-2 pandemic has accomplished worldwide relevance during the last year, and coinfections with *S. pneumoniae* are associated with poor prognosis and outcomes.^42^ Developing tests for the detection of *S. pneumoniae* is urgently needed, especially in children, because urinary antigen tests used in adults cannot discern between sick and asymptomatic carrier children. On the other hand, spreading of antibiotic-resistant strains is growing worryingly due to the use of broad-spectrum antibiotics before the pathogen can be accurately detected.

The LAMP procedure here developed is based on one previously reported^43^ that uses PR as a visual indicator. To decrease false negative results that could be indistinguishable by naked eye detection, we propose to take advantage of the differences of the PR redox process with pH. On this basis, once the LAMP is performed, the amplification solution is deposited on a screen-printed low-volume electrochemical cell (≈20 μL) for the measurement. This setup favors the portability of the test allowing quantitative results with improvement of sensitivity.

For the rapid diagnosis of infectious diseases with POC platforms, non-invasive samples are preferred, as for example, nasopharyngeal exudate, sputum, urine, and even saliva as it is already being done for COVID-19 rapid testing.^44,45^ Regarding pneumococcal pneumonia, it has been proven that the ply gene can be detected in urine samples.^46,47^ In this way, the electrochemical LAMP here developed can be applied to ply detection in urine samples, which is very convenient from the point of view of non-invasiveness.

## Experimental Section

All chemicals were of analytical reagent grade and provided by Sigma-Aldrich, and for amplification experiments, ultrapure DNase and RNase free water was used.

### Preparation of DNA

The pTrc99A-ply plasmid,^48^ containing the ply gene, was extracted using a Qiagen-tip anion-exchange column (Qiagen). DNA was quantified in an Ultrospec 3300 Pro spectrophotometer (Amersham Pharmacia Biotech).

### LAMP assay

Based on the sequence of *S. pneumoniae* strain R6 (GenBank AE008540), four ply-specific LAMP primers were designed using LAMP primer support design software (PrimerExplorer v4; Eiken Chemical Ltd) to amplify a 175-bp fragment, as previously described.^48^ The reaction mix using WarmStart Colorimetric LAMP 2X Master Mix (DNA & RNA) (New England Biolabs) contained 1 × master mix (include Bst 2.0 DNA polymerase), 0.7 mM dUTG, primer mix (1.6 μM each FIP and BIP, 0.4 μM each of ply-F3 and ply-B3 primer), 0.3 U of Antarctic Thermolabile UDG (Uracil-DNA glycosylase) (New England Biolabs) and template DNA. Samples containing RNase-DNase free H_2_O were used as negative controls. The mixes were incubated at 37 °C for 30 min followed by incubation at 65 °C for 90 min and then warmed at 80 °C for 2 min to stop the reaction in a PCR machine (Veriti 96 Well Thermal Cycler, Applied Biosystems). For confirmation of DNA amplification, LAMP reaction products were resolved by electrophoresis on 3% agarose gels and stained with ethidium bromide.

### Electrochemical Measurements

All electrochemical measurements were performed at room temperature using thick-film carbon electrodes (S1PE, MicruX Technologies) connected to a μAUTOLAB TYPE III (Metrohm) potentiostat through a BOX Connector (ED-SPE-BOX, MicruX Technologies).

For measurements at different pH values ranging from 4.0 to 10.0, Britton Robinson (BR) buffer solutions were used.

To obtain analytical signals that can be correlated with the initial concentration of DNA, a volume of 20 μL of end-point LAMP reactions was deposited onto each electrochemical cell one by one.

For both, the study of PR electrochemical behavior and its detection after LAMP reactions, cyclic voltammograms (CVs) were recorded scanning the potential between −0.5 and + 1.0 V at a scan rate of 100 mV·s^–1^. Alternatively, linear sweep voltammograms (LSVs) were obtained by scanning the potential only in the positive direction, between 0.1 and + 1.0 V at 100 mV·s^–1^, to record the anodic process of PR.

### Urine Sample collection and Treatment

Urine samples were obtained from 10 healthy children at a healthcare center in Lugones (Asturias, Spain) during routine pediatric check-ups for DNA spiking experiments. Children were considered healthy if they did not show respiratory symptoms, had not received any antibiotic treatment during the previous week, and had not been hospitalized for any reason during the previous month. Samples were frozen at −70 °C until analysis. After thawing overnight at 8 °C, a volume of 20 mL of each sample was centrifuged at 3000 *g* for 10 min, and the supernatant was transferred to a new tube. The pH of samples was adjusted conveniently, and 1 mL of the supernatant was centrifuged at 15 000 *g* for 10 min, transferring the supernatant to a new tube. This study was carried out in accordance with the recommendations of the Ethical Committee on Regional Clinical Research of the Principality of Asturias.

## Results and Discussion

### Sensitivity of LAMP performed in Microcentrifuge Tubes

**Figure 1:**
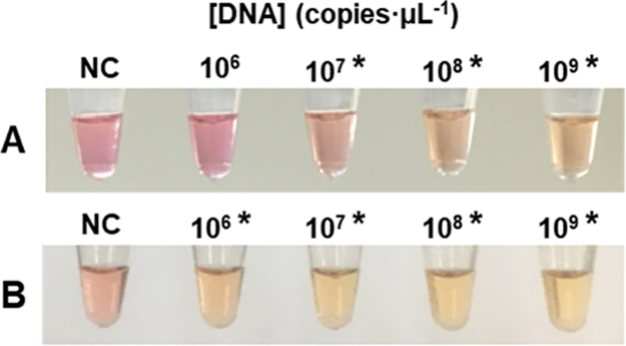
Visual LAMP reaction products of ply gene. End-point LAMP reaction products in (A) RNase-DNase free H_2_O and (B) spiked urine samples. NC: negative control; *positive LAMP reactions detected by the naked eye.

In order to prevent carryover contamination, dUTG and Antarctic Thermolabile UDG were added to the colorimetric master mix.^49^ The sensitivity of the *S. pneumoniae* LAMP assay was evaluated using 10 × dilution series ranging from 10^9^ to 10^6^ copies·μL^–1^ of plasmid DNA template pTrc99A-ply. Amplification of the target sequence (ply) was performed using a four-primer set previously designed.^43^ All LAMP reactions were performed in triplicate. Negative controls were performed with 1 μL of RNase-DNase free water. After 90 min of incubation at 65 °C, LAMP reactions were visually read with a limit of detection (LOD) of about 10^7^ copies·μL^–1^ (Figure 1A).

### DNA spiking in urine Samples

A pool of 10 urine samples of healthy children were used for DNA spiking experiments. DNA template pTrc99A-ply and untreated urine were mixed to a final DNA concentration of 10^9^ copies·μL^–1^ in a volume of 100 μL, and serial 10-fold dilutions of pTrc99A-ply ranging from 10^9^ to 10^6^ copies·μL^–1^ were made. For LAMP reactions, 1 μl of each dilution was used as the DNA template. We observed that addition of template to the mix reaction changed all tubes to yellow and, after incubation, no amplification was observed. After treatment of samples to eliminate salts and adjusting pH to 7.0, new 10-fold dilution series were made using treated urine. Negative controls were performed with 1 μL of treated urine. The results obtained after 90 min of LAMP reaction are shown in Figure 1B.

### Electrochemical Behavior of PR

**Figure 2:**
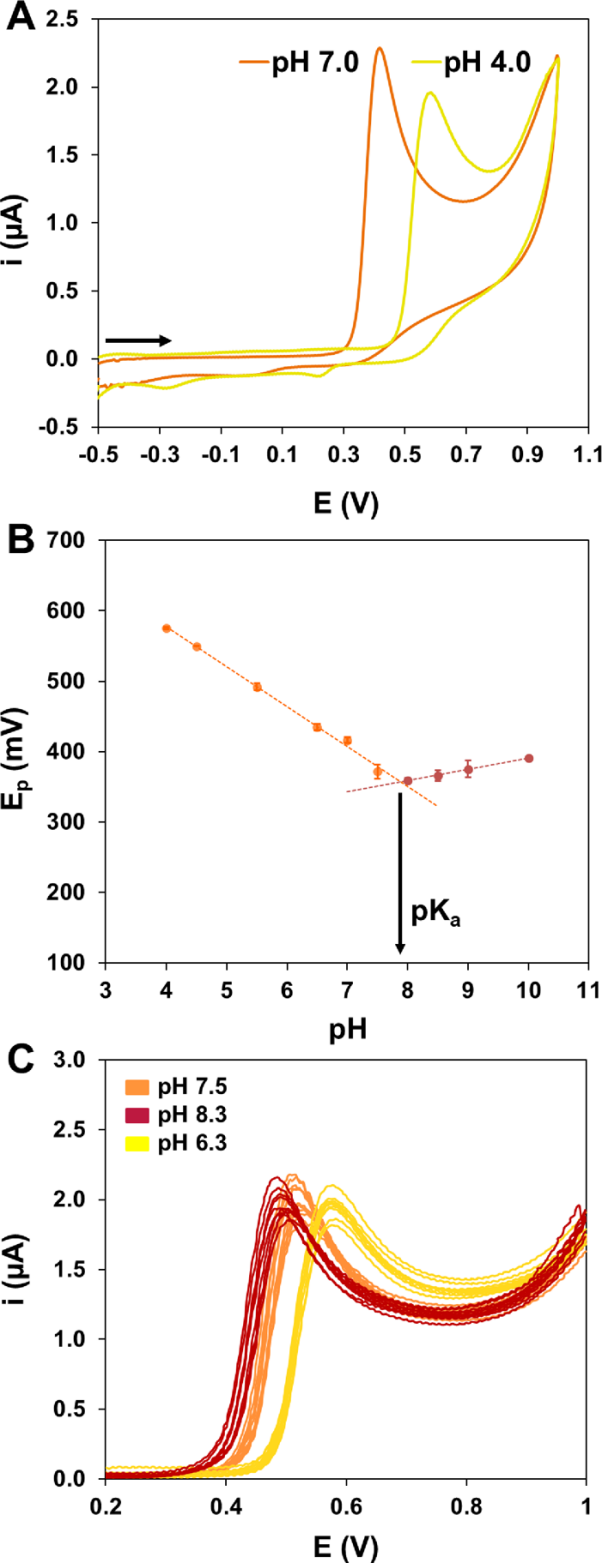
(A) CVs of 0.1 mM solutions of PR in BR buffer solutions of pH 7.0 and 4.0. (B) PR oxidation peak potential versus pH from pH 4.0 to 8.0 (orange) and from pH 8.0 to 10.0 (red); the p*K*_a_ of PR is the pH value which corresponds to the intersection between both lines; error bars correspond to the standard deviation of three measurements. (C) LSVs recorded on 10 different electrodes in 0.1 mM PR solutions of different pH values.

PR is the simplest form of sulfonephthaleins with three aromatic rings containing a quinone methide group with redox properties^37^ (Figure S1). To study its electrochemical behavior in the screen-printed electrodes that are going to be used for LAMP detection, CVs were recorded in 0.1 mM solutions of PR in BR buffer solutions of different pH values ranging from 4.0–10.0. The potential was scanned from –0.5 to + 1.0 V at a rate of 100 mV·s^–1^. Figure 2A shows the voltammograms corresponding to pH 4.0 and 7.0. This pH indicator presents a main irreversible anodic process at both pH values, with higher potential for the lower pH value. A non-well-defined anodic process, more noticeable at low pH, appears at higher potentials. These unconnected first and second anodic processes correspond to the oxidation of unprotonated and protonated PR forms,^37^ with a radical cation resulting as a product. Cathodic processes of low intensity are also observed in this potential window, again more noticeable at lower pH. Since the main oxidation process is the only one here considered useful for analytical purposes, the backward scan will not be recorded, decreasing the time of measurement to the half. Thus, LSVs will be recorded instead. In Figures S2B and S2C the LSVs for PR recorded in BR solutions with pH values ranging from 4.0 to 8.0 and from 8.0 to 10.0 are presented. The values of peak potentials and peak currents are reported in Table S1. It can be clearly observed that the anodic process moves to less positive values with increasing pH, from 4.0 to 8.0; meanwhile, it moves back toward more positive values when increasing to 10.0. This can be quantitatively observed in the graph of Figure 2B that represents the anodic peak potential versus pH, showing two linear ranges.

The equations are *E*_p_ (mV) = −56.6 pH + 803.8 (*R*^2^ = 0.996, *n* = 6) between 4.0 and 7.5 and *E*_p_ (mV) = 16.1 pH + 229.9 (*R*^2^ = 0.998, *n* = 4) between 8.0 and 10.0. The lines intersect at pH 7.9 that corresponds to the p*K*_a_ of the indicator, which is in concordance with that reported in the bibliography.^50,51^ The value of the slope at pH values lower than p*K*_a_ (close to the theoretical Nernstian) indicates that the same number of electrons and protons are involved in the anodic process. The pH interval of color change corresponds to the pH range of the LAMP reaction (Figure S2A), resulting PR an adequate indicator; moreover, pH changes in LAMP reaction correspond to the pH range in which PR peak potential variations show a higher slope. Thus, it ranges from *ca.* pH 8.0 (no DNA amplification, negative result) to *ca.* pH 6.5 (DNA amplification, positive result).^52^ Results observed in Figure 2 suggest that DNA could be monitored by variations in the potential of the anodic peak of PR, the visual indicator, at the end of LAMP reactions.

Cyclic voltammetry (or linear sweep voltammetry) is a diagnostic technique employed initially to know the electrochemical behavior of species. Once the process of interest has been chosen, other techniques (e.g., pulse techniques such as square wave voltammetry) can be employed to decrease the LOD. However, since a linear scan is employed in cyclic voltammetry/linear sweep voltammetry, the electronics of the instrumentation is simpler, which is important to not increase the cost and the complexity of future devices and procedures, aimed to decentralize diagnostics. Moreover, since the PR process is not reversible, the increase in sensitivity produced when using pulse techniques is not expected to be notorious.

With the aim of reusing the same electrode for different measurements, it was explored if recording successive CVs on the same electrode affected the signal (Figure S3). When reaction intermediates or products adsorb, active sites on the electrode may be blocked. At the very least, molecular adsorption creates a prior history that can be carried over to the next measurement if the electrode is not properly cleaned.^53^ In this case, a decrease in the peak current and a movement in the potential of the PR process, in concordance with this reported in the bibliography,^9,11^ is seen. Consecutive CVs of PR form polymeric films on the surface of the electrode, which affect its ability for electronic transference but generate possible mediators for other electron transfer processes. Actually, the modification of glassy carbon electrodes with conductive poly(PR) is employed for acetaminophen and dopamine^37^ or lead (II)^54^ determinations. However, to have a reproducible initial electrode surface, the electrochemical measurement after each LAMP reaction was done with a new electrode to assure the precision of the potential readout. A pseudoreference electrode does not present the stability of traditional reference electrodes. However, screen-printed electrochemical cells are thought as single-use transducers, as in this case. Therefore, the interelectrode precision is more important than the long-term stability. With the aim of evaluating it, voltammograms were recorded on 10 electrodes in a 0.1 mM solution of PR (Tris-HCl buffer solutions) at different pH values (to simulate the change during LAMP reactions). The values of the anodic peak potential of PR were 493 ± 7, 521 ± 5, and 576 ± 3 mV at pH 8.30, 7.50, and 6.35, respectively. Values for RSD were lower than 1.4% in all cases. Figure 2C represents the corresponding LSVs.

### Electrochemical Detection of LAMP Reactions

**Figure 3:**
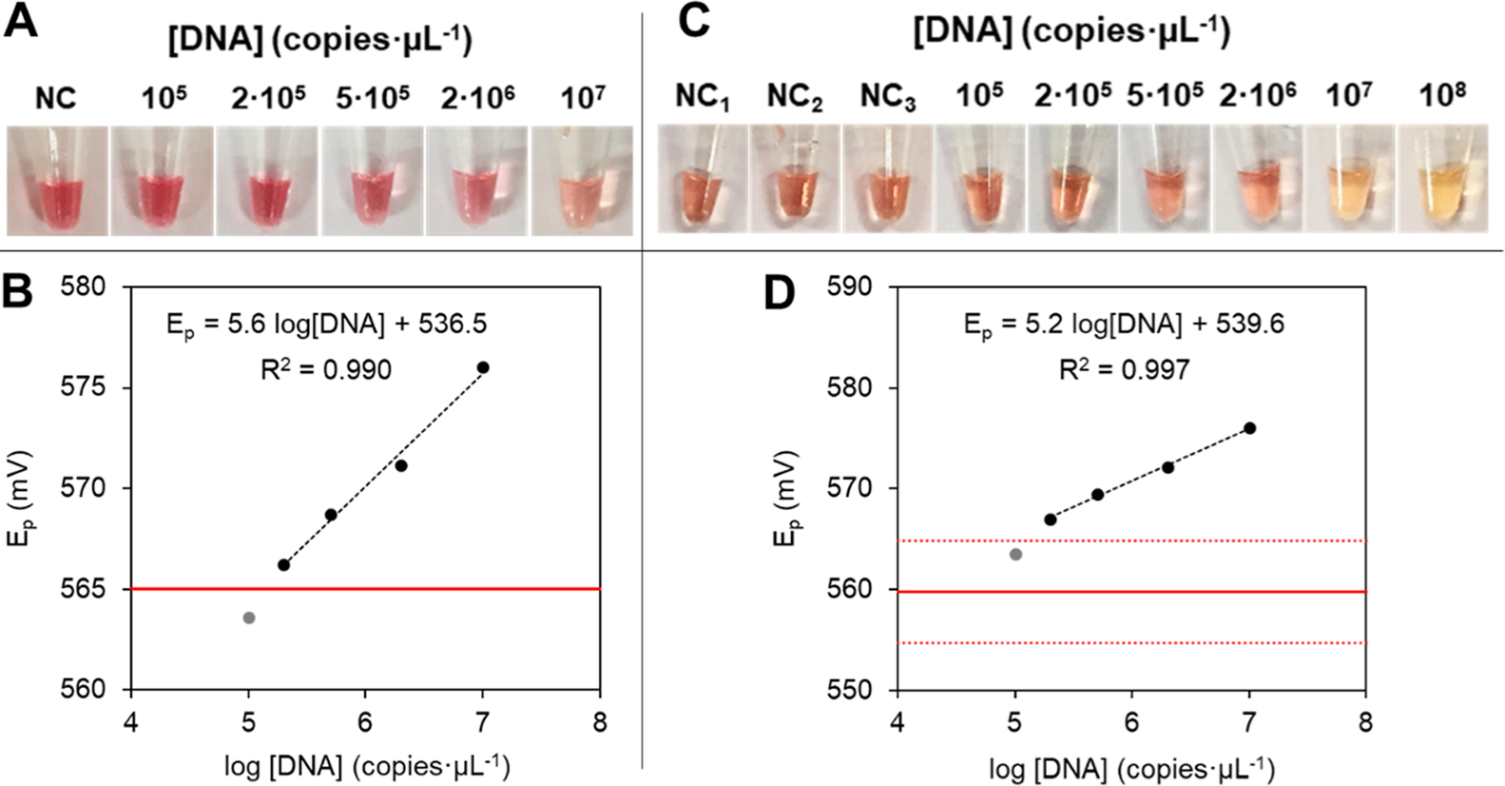
(A) End-point LAMP products obtained using different initial DNA dilutions (copies·μL^–1^ are shown in figure). A negative control (NC) is also shown. (B) Calibration plot using electrochemical LAMP detection for the reactions shown in (A). The peak potential value for NC is represented as a continuous red line. The value for 10^5^ copies·μL^–1^ is shown in gray. The equation of the linear relationship is included as an inset. (C) End-point LAMP products obtained in urine spiked samples and three negative controls. (D) Calibration plot using electrochemical LAMP for the reactions shown in (C). The mean peak potential value for NCs is represented as a continuous red line, with the values for ± SD represented as dashed red lines. The value for the addition of 10^5^ copies·μL^–1^ is shown in gray. The equation for the linear relationship is included as an inset.

Figure 3A presents the products of end-point LAMP reactions performed using different initial DNA dilutions in water that ranged from 10^7^ to 10^5^ copies·μL^–1^. As it can be seen, the tubes with concentrations ranging from 10^5^ to 2 × 10^6^ copies·μL^–1^ would be considered negative based on visual colorimetric detection. However, as seen in Figure 3B, the displacement in the potential of the anodic peak after LAMP reaction showed a linear relationship with the logarithm of the concentration up to 10^7^ copies·μL^–1^, with an *R*^2^ of 0.990, following the equation: *E*_p_ (mV) = 5.6 log [DNA](copies·μL^–1^) + 536.5.

Figure S4 shows the corresponding box and whiskers plot for 10 negative and 10 positive amplification reactions (i.e., containing 0 and 2 × 10^5^ copies·μL^–1^). A significant difference between negative and positive amplifications is seen. Atypical values were not detected. Both medians were slightly closer to the first quartile. The interquartile range, which represents the 50% of the results, was slightly smaller for negative values. The maximum value for negative reactions was −0.587 V; meanwhile the minimum for the positive was −0.589 V, with no overlap of whiskers. Since the signals corresponding to 2 × 10^5^ copies·μL^–1^ are clearly discernible from the negative control signal, we consider this value as the practical limit of detection (LOD). Nevertheless, a set of negative reactions must be always carried out because variations between different lots of the RT-LAMP kit, which include biological reagents, or slight differences in the potential measured using screen-printed carbon electrodes of different batches can occur.

Therefore, this novel approach allows qualitative-to-quantitative conversion of the methodology. In the qualitative analysis, sensitivity is defined as the ratio between true positive and total positive values, being the last addition of true positive and false negative values. Considering that a negative result would have been assigned to concentrations comprised between 10^5^ and 2 × 10^6^ copies·μL^–1^, being positive, the use of this simple ED that can be performed with low-cost and decentralizable methodology would allow to increase the sensitivity considerably. The measurement takes 26 s (scan between + 0.2 and + 0.9 V at a scan rate of 100 mV·s^–1^), a value that does not increase the sample-to-result time significantly.

As a comparison, the color presented after LAMP reactions has also been measured from captured images analyzed using the open-source image processing software ImageJ.^55^ Results are included in Figure S5. Images were first RGB split and then measured in the green channel, as it has been previously reported for LAMP.^56^ The linear relationship between the intensity of green and the logarithm of the concentration of DNA in copies·μL^–1^ follows the equation *I*_G_ = 35.4 log [DNA] −117 (*R*^2^ = 0.998). Although the sensitivity is higher, 2 × 10^5^ copies·μL^–1^ cannot be distinguished from the negative control. Apart from this, adequate image capture and treatment are required. However, detection by the naked eye of PR, indicator that is included in the kit, is a confirmation of positive results that can be further quantified electrochemically.

### Application to Urine Sample analysis

Results obtained with LAMP reactions for standard DNA solutions (in water) were very promising. Then, we intended to evaluate this fast and simple methodology for urine sample analysis. The presence of whole *S. pneumoniae* is never expected in urine samples from patients with pneumococcal pneumonia since only cell-free DNA could cross the renal barrier. However, microorganisms that cause urinary tract infections could be present. Therefore, we have previously studied the specificity of the primers.^43^ We aimed to assess the capacity of the methodology to reduce false negative results provided by colorimetric detection. For this study, nine urine samples comprising three negative controls and six spiked urines to obtain 10^5^, 2 × 10^5^, 5 × 10^5^, 2 × 10^6^, 10^7^, and 10^8^ copies·μL^–1^ were electrochemically measured after LAMP reactions as indicated above. Figure 3C shows the end-point LAMP reaction products. As can be seen, only the first two more concentrated samples can be confidently considered positive by visual inspection. Although a more thorough study is required, it can be said that after electrochemical measurement of each sample, a cutoff value (mean peak potential for negative controls + SD), could be established. Results in Figure 3D confirm that the samples with dilutions ranging from 2 × 10^5^ to 2 × 10^6^ copies·μL^–1^ can also be considered positive, apart from those corresponding to 10^7^ and 10^8^ copies·μL^–1^, that can be assigned positive by naked eye detection, while the dilution with 10^5^ copies·μL^–1^ is below the cutoff. This dilution (i.e., 2 × 10^5^ copies·μL^–1^) could be considered the LOD of this method.

Other ply gene detection methods previously described are based on PCR assays that require expensive apparatus, specialized personnel, and a minimum development time superior to the LAMP method. The LOD of the electrochemical LAMP methodology is similar (or even lower) to that obtained with color measurement. A report in the literature describes a ply-LAMP amplification with fluorescent detection limit of 300 pg·μL^–1^ (i.e., ca. 1.3 × 10^5^ copies·μL^–1^).^57^ The detection of LAMP reactions with agarose gel electrophoresis decreases the LOD to 10^2^–10^3^ copies·μL^–1^, although decentralization is not possible. The sensitivity of our methodology seems to be sufficient for the detection of *S. pneumoniae* DNA in urine samples.^43^ However, this does not prevent us from addressing strategies of sensitivity improvement when we have sufficient clinical samples.

The equation for the relationship between *E*_p_ and DNA concentration is *E*_p_ (mV) = 5.2 log [DNA](copies·μL^–1^) + 539.6, *R*^2^ = 0.997. Comparing the slope obtained for both matrices (Figure 3B,D), a *t*_cal_ of 0.0647 is obtained, and considering a coverage factor (*k*) of 2 and a 95% level of confidence, it can be said that there are no significant differences and therefore matrix effects. As in the case of LAMP performed using DNA diluted in water, a practical LOD of 2 × 10^5^ copies·μL^–1^ can be considered. The differences between visual and ED of LAMP reactions allow us to consider this methodology very promising, especially at low concentration levels where confirmatory analysis is required. Therefore, work is in progress to apply the methodology to the analysis of urine of children with symptoms of CAP and to adapt the methodology to the detection of other infectious pathogens.

## Conclusions

Infectious diseases require rapid information for patient care allowing accurate antimicrobial treatment. Failing in the diagnosis usually means failing in the treatment. When providing a result, being able of certainly discriminating between who is really affected by the disease and who is not is of great importance. Decreasing false negative results is a challenge that must be urgently faced, and developing low-LOD tests is the way. In this work, a method for electrochemical detection of isothermal amplifications of ply gene was developed using PR as the indicator, obtaining a practical LOD of 2 × 10^5^ copies·μL^–1^, not appreciable by the naked eye.

An innovative electrochemical approach was used to improve considerably the sensitivity, demonstrating its ability to detect positive amplification in visual negative samples. With a simple and fast measurement using miniaturized and low-cost electrodes that require very low volume of solution, the LOD of LAMP was decreased considerably, from 2 × 10^6^ to 2 × 10^5^ copies·μL^–1^. This was maintained in urine samples spiked with pneumococcal DNA. Moreover, urine treatment is minimal compared with DNA extraction procedures previously reported.^43^ Although there are some LAMP-ED methodologies reported, this is the first time that, to the best of our knowledge, electrochemical detection of PR is used for the sensitive detection of LAMP reactions. A highly sensitive detection of the ply gene of *S. pneumoniae* was achieved by monitoring the change in the peak potential of the redox process of PR.

Although further studies should be made to maximize the potential of the LAMP-ED combination, as well as thorough clinical studies, this work opens a new way to improve the diagnosis of infectious diseases. The possibilities of decentralization and the decrease in the analysis time make this methodology highly desirable for being prepared in case future microbiological threats appear, as was the case of the current pandemic situation.

## Supporting Information Available

Chemical structure of PR; visual and electrochemical PR behavior at different pH values; successive CVs of PR on the same electrode; box and whiskers plot with the values for the PR peak potential values in LAMP reactions; calibration curves obtained using the intensity of the green (Image J software) (PDF)

## References

[ref1] Udugama B.; Kadhiresan P.; Kozlowski H. N.; Malekjahani A.; Osborne M.; Li V. Y. C.; Chen H.; Mubareka S.; Gubbay J. B.; Chan W. C. W. Diagnosing COVID-19: The Disease and Tools for Detection. ACS Nano 2020, 14, 3822–3835. 10.1021/acsnano.0c02624. 32223179

[ref2] Liu J.; Geng Z.; Fan Z.; Liu J.; Chen H. Point-of-care testing based on smartphone: The current state-of-the-art (2017-2018). Biosens. Bioelectron. 2019, 132, 17–37. 10.1016/j.bios.2019.01.068. 30851493

[ref3] Riccò M.; Ferraro P.; Gualerzi G.; Ranzieri S.; Henry B. M.; Said Y. Ben.; Pyatigorskaya N. V.; Nevolina E.; Wu J.; Bragazzi N. L.; Signorelli C. Point-of-Care Diagnostic Tests for Detecting SARS-CoV-2 Antibodies: A Systematic Review and Meta-Analysis of Real-World Data. J. Clin. Med. 2020, 9, 1515 10.3390/jcm9051515. PMC7290955 32443459

[ref4] Trinh T. N. D.; Lee N. Y. A Rapid and Eco-Friendly Isothermal Amplification Microdevice for Multiplex Detection of Foodborne Pathogens. Lab Chip 2018, 18, 2369–2377. 10.1039/c8lc00424b. 29923578

[ref5] Petralia S.; Conoci S. PCR Technologies for Point of Care Testing: Progress and Perspectives. ACS Sens. 2017, 2, 876–891. 10.1021/acssensors.7b00299. 28750519

[ref6] Zhu H.; Zhang H.; Ni S.; Korabečná M.; Yobas L.; Neuzil P. The Vision of Point-of-Care PCR Tests for the COVID-19 Pandemic and Beyond. TrAC, Trends Anal. Chem. 2020, 130, 115984 10.1016/j.trac.2020.115984. PMC7369599 32834243

[ref7] Zhao Y.; Chen F.; Li Q.; Wang L.; Fan C. Isothermal Amplification of Nucleic Acids. Chem. Rev. 2015, 115, 12491–12545. 10.1021/acs.chemrev.5b00428. 26551336

[ref8] Notomi T.; Mori Y.; Tomita N.; Kanda H. Loop-Mediated Isothermal Amplification (LAMP): Principle, Features, and Future Prospects. J. Microbiol. 2015, 53, 1–5. 10.1007/s12275-015-4656-9. 25557475

[ref9] Tanner N. A.; Zhang Y.; Evans T. C. Visual Detection of Isothermal Nucleic Acid Amplification Using PH-Sensitive Dyes. Biotechniques 2015, 58, 59–68. 10.2144/000114253. 25652028

[ref10] Kampeera J.; Pasakon P.; Karuwan C.; Arunrut N.; Sappat A.; Sirithammajak S.; Dechokiattawan N.; Sumranwanich T.; Chaivisuthangkura P.; Ounjai P.; Chankhamhaengdecha S.; Wisitsoraat A.; Tuantranont A.; Kiatpathomchai W. Point-of-Care Rapid Detection of Vibrio Parahaemolyticus in Seafood Using Loop-Mediated Isothermal Amplification and Graphene-Based Screen-Printed Electrochemical Sensor. Biosens. Bioelectron. 2019, 132, 271–278. 10.1016/j.bios.2019.02.060. 30878727

[ref11] Olabarria G.; Eletxigerra U.; Rodriguez I.; Bilbao A.; Berganza J.; Merino S. Highly sensitive and fast Legionella spp. in situ detection based on a loop mediated isothermal amplification technique combined to an electrochemical transduction system. Talanta 2020, 217, 121061 10.1016/j.talanta.2020.121061. 32498831

[ref12] Reuter C.; Slesiona N.; Hentschel S.; Aehlig O.; Breitenstein A.; Csáki A.; Henkel T.; Fritzsche W. Loop-Mediated Amplification as Promising on-Site Detection Approach for Legionella Pneumophila and Legionella Spp. Appl. Microbiol. Biotechnol. 2020, 104, 405–415. 10.1007/s00253-019-10286-3. 31832709

[ref13] Cai Z.-H.; Dai Y.-Y.; Huang L.-Y.; Zhang W.-S.; Guo X.-G. Diagnosis of Mycoplasma Pneumoniae by Loop-Mediated Isothermal Amplification: Systematic Review and Meta-Analysis. BMC Infect. Dis. 2019, 19, 173 10.1186/s12879-019-3799-4. 30782134 PMC6379949

[ref14] Vergara A.; Boutal H.; Ceccato A.; López M.; Cruells A.; Bueno-Freire L.; Moreno-Morales J.; Puig de la Bellacasa J. P. de la.; Castro P.; Torres A.; Marco F.; Casals-Pascual C.; Vila J. Assessment of a Loop-Mediated Isothermal Amplification (LAMP) Assay for the Rapid Detection of Pathogenic Bacteria from Respiratory Samples in Patients with Hospital-Acquired Pneumonia. Microorganisms 2020, 8, 103 10.3390/microorganisms8010103. PMC7022425 31940771

[ref15] Kashir J.; Yaqinuddin A. Loop Mediated Isothermal Amplification (LAMP) Assays as a Rapid Diagnostic for COVID-19. Med. Hypotheses 2020, 141, 109786 10.1016/j.mehy.2020.109786. 32361529 PMC7182526

[ref16] Mohon A. N.; Oberding L.; Hundt J.; van Marle G. van; Pabbaraju K.; Berenger B. M.; Lisboa L.; Griener T.; Czub M.; Doolan C.; Servellita V.; Chiu C. Y.; Greninger A. L.; Jerome K. R.; Pillai D. R. Optimization and Clinical Validation of Dual-Target RT-LAMP for SARS-CoV-2. J. Virol. Methods 2020, 286, 113972 10.1016/j.jviromet.2020.113972. 32941977 PMC7490281

[ref17] Rödel J.; Egerer R.; Suleyman A.; Sommer-Schmid B.; Baier M.; Henke A.; Edel B.; Löffler B. Use of the variplex SARS-CoV-2 RT-LAMP as a rapid molecular assay to complement RT-PCR for COVID-19 diagnosis. J. Clin. Virol. 2020, 132, 104616 10.1016/j.jcv.2020.104616. 32891938 PMC7457909

[ref18] da Silva S. J. R.; Pardee K.; Pena L. Loop-Mediated Isothermal Amplification (LAMP) for the Diagnosis of Zika Virus: A Review. Viruses 2019, 12, 19 10.3390/v12010019. PMC7019470 31877989

[ref19] Ahn S. J.; Baek Y. H.; Lloren K. K. S.; Choi W.-S.; Jeong J. H.; Antigua K. J. C.; Kwon H.; Park S.-J.; Kim E.-H.; Kim Y.; Si Y.-J.; Hong S. B.; Shin K. S.; Chun S.; Choi Y. K.; Song M.-S. Rapid and simple colorimetric detection of multiple influenza viruses infecting humans using a reverse transcriptional loop-mediated isothermal amplification (RT-LAMP) diagnostic platform. BMC Infect. Dis. 2019, 19, 676 10.1186/s12879-019-4277-8. 31370782 PMC6669974

[ref20] Wang D.; Yu J.; Wang Y.; Zhang M.; Li P.; Liu M.; Liu Y. Development of a real-time loop-mediated isothermal amplification (LAMP) assay and visual LAMP assay for detection of African swine fever virus (ASFV). J. Virol. Methods 2020, 276, 113775 10.1016/j.jviromet.2019.113775. 31726114

[ref21] Wang N.; Pan G.; Guan S.; Rong S.; Wang D.; Gao Z.; Tian P.; Li Q. A Broad-Range Disposable Electrochemical Biosensor Based on Screen-Printed Carbon Electrodes for Detection of Human Noroviruses. Front. Bioeng. Biotechnol. 2022, 10, 1–9. 10.3389/fbioe.2022.845660. PMC8992780 35402404

[ref22] Jaroenram W.; Kampeera J.; Arunrut N.; Karuwan C.; Sappat A.; Khumwan P.; Jaitrong S.; Boonnak K.; Prammananan T.; Chaiprasert A.; Tuantranont A.; Kiatpathomchai W. Graphene-Based Electrochemical Genosensor Incorporated Loop-Mediated Isothermal Amplification for Rapid on-Site Detection of Mycobacterium Tuberculosis. J. Pharm. Biomed. Anal. 2020, 186, 113333 10.1016/j.jpba.2020.113333. 32402994

[ref23] Fu Y.; Zhou X.; Duan X.; Liu C.; Huang J.; Zhang T.; Ding S.; Min X. A LAMP-Based Ratiometric Electrochemical Sensing for Ultrasensitive Detection of Group B Streptococci with Improved Stability and Accuracy. Sensors and Actuators, B: Chemical 2020, 321, 128502 10.1016/j.snb.2020.128502.

[ref24] Nagatani N.; Yamanaka K.; Saito M.; Koketsu R.; Sasaki T.; Ikuta K.; Miyahara T.; Tamiya E. Semi-Real Time Electrochemical Monitoring for Influenza Virus RNA by Reverse Transcription Loop-Mediated Isothermal Amplification Using a USB Powered Portable Potentiostat. Analyst 2011, 136, 5143–5150. 10.1039/c1an15638a. 22010112

[ref25] Ramírez-Chavarría R. G.; Castillo-Villanueva E.; Alvarez-Serna B. E.; Carrillo-Reyes J.; Ramírez-Zamora R. M.; Buitrón G.; Alvarez-Icaza L. Loop-Mediated Isothermal Amplification-Based Electrochemical Sensor for Detecting SARS-CoV-2 in Wastewater Samples. J. Environ. Chem. Eng. 2022, 10, 107488 10.1016/j.jece.2022.107488. 35251932 PMC8883760

[ref26] Tamiya E. Portable Electrochemical DNA Sensors Based on Gene Amplification Reactions to Screen and Identify Pathogen and SNPs. Sensors 2022, 22, 1865 10.3390/s22051865. 35271014 PMC8914808

[ref27] Safavieh M.; Ahmed M. U.; Ng A.; Zourob M. High-Throughput Real-Time Electrochemical Monitoring of LAMP for Pathogenic Bacteria Detection. Biosens. Bioelectron. 2014, 58, 101–106. 10.1016/j.bios.2014.02.002. 24632135

[ref28] Anton M.; Moranova L.; Hrstka R.; Bartosik M. Application of an Electrochemical LAMP-Based Assay for Screening of HPV16/HPV18 Infection in Cervical Samples. Anal. Methods 2020, 12, 822–829. 10.1039/c9ay02383f.

[ref29] Xie S.; Yuan Y.; Chai Y.; Yuan R. Tracing Phosphate Ions Generated during Loop-Mediated Isothermal Amplification for Electrochemical Detection of Nosema Bombycis Genomic DNA PTP1. Anal. Chem. 2015, 87, 10268–10274. 10.1021/acs.analchem.5b01858. 26412581

[ref30] Chand R.; Wang Y. L.; Kelton D.; Neethirajan S. Isothermal DNA amplification with functionalized graphene and nanoparticle assisted electroanalysis for rapid detection of Johne’s disease. Sensors and Actuators, B: Chemical 2018, 261, 31–37. 10.1016/j.snb.2018.01.140.

[ref31] Hua X.; Yang E.; Yang W.; Yuan R.; Xu W. LAMP-Generated H+ Ions-Induced Dimer i-Motif as Signal Transducer for Ultrasensitive Electrochemical Detection of DNA. Chem. Commun. 2019, 55, 12463–12466. 10.1039/c9cc06738h. 31576854

[ref32] Zhao J.; Gao J.; Zheng T.; Yang Z.; Chai Y.; Chen S.; Yuan R.; Xu W. Highly Sensitive Electrochemical Assay for Nosema Bombycis Gene DNA PTP1 via Conformational Switch of DNA Nanostructures Regulated by H+ from LAMP. Biosens. Bioelectron. 2018, 106, 186–192. 10.1016/j.bios.2018.02.003. 29427924

[ref33] Thu V. T.; Tien B. Q.; Ngoc Nga D. T.; Thanh L. C.; Sinh L. H.; Le T. C.; Lam T. D. Reduced Graphene Oxide-Polyaniline Film as Enhanced Sensing Interface for the Detection of Loop-Mediated-Isothermal-Amplification Products by Open Circuit Potential Measurement. RSC Adv. 2018, 8, 25361–25367. 10.1039/c8ra04050h. 35539802 PMC9082585

[ref34] Kong H.; Zhang W.; Yao J.; Li C.; Lu R.; Guo Z.; Li J.; Li C.; Li Y.; Zhang C.; Zhou L. A RT-LAMP Based Hydrogen Ion Selective Electrode Sensing for Effective Detection HIV-1 RNA with High-Sensitivity. Sensors and Actuators, B: Chemical 2021, 329, 129118 10.1016/j.snb.2020.129118.

[ref35] Lee K. H.; Lee D.; Yoon J.; Kwon O.; Lee J. A Sensitive Potentiometric Sensor for Isothermal Amplification-Coupled Detection of Nucleic Acids. Sensors 2018, 18, 2277 10.3390/s18072277. PMC6068556 30011898

[ref36] Senne J. K.; Marple L. W. Electrochemical Reduction of Phenol Red. Anal. Chem. 1970, 42, 1147–1150. 10.1021/ac60293a008.

[ref37] Hsieh M. T.; Whang T. J. Mechanistic Investigation on the Electropolymerization of Phenol Red by Cyclic Voltammetry and the Catalytic Reactions toward Acetaminophen and Dopamine Using Poly(Phenol Red)-Modified GCE. J. Electroanal. Chem. 2017, 795, 130–140. 10.1016/j.jelechem.2017.05.001.

[ref38] Karabiberoğlu Ş. U.; Dursun Z. Over-Oxidized Poly (Phenol Red) Film Modified Glassy Carbon Electrode for Anodic Stripping Voltammetric Determination of Ultra-Trace Antimony (III). Electroanalysis 2017, 29, 1069–1080. 10.1002/elan.201600629.

[ref39] Tilley S. J.; Orlova E. V.; Gilbert R. J. C.; Andrew P. W.; Saibil H. R. Structural Basis of Pore Formation by the Bacterial Toxin Pneumolysin. Cell 2005, 121, 247–256. 10.1016/j.cell.2005.02.033. 15851031

[ref40] Mongardon N.; Max A.; Bouglé A.; Pène F.; Lemiale V.; Charpentier J.; Cariou A.; Chiche J. D.; Bedos J. P.; Mira J. P. Epidemiology and Outcome of Severe Pneumococcal Pneumonia Admitted to Intensive Care Unit: A Multicenter Study. Crit. Care 2012, 16, R155 10.1186/cc11471. 22894879 PMC3580745

[ref41] Cillóniz C.; Nicolini A.; Luque N.; Torres A. Community-Acquired Pneumonia and Acute Respiratory Distress Syndrome: Prevalence, Risk, and Prognosis. Clin. Pulm. Med. 2018, 25, 100–106. 10.1097/CPM.0000000000000262.

[ref42] Feldman C.; Anderson R. The Role of Co-Infections and Secondary Infections in Patients with COVID-19. Pneumonia 2021, 13, 15 10.1186/s41479-021-00083-w. PMC8068564 33894790

[ref43] Cima-Cabal M. D.; Vázquez-Espinosa E.; Vazquez F.; García-Suárez M. D. M. Detection of Streptococcus Pneumoniae in Urine by Loop-Mediated Isothermal Amplification. J. Pediatr. Infect. Dis. 2021, 16, 018–025. 10.1055/s-0040-1719164.

[ref44] Hamid H.; Khurshid Z.; Adanir N.; Zafar M. S.; Zohaib S. Covid-19 Pandemic and Role of Human Saliva as a Testing Biofluid in Point-of-Care Technology. Eur. J. Dent. 2020, 14, S123 10.1055/S-0040-1713020. 32492721 PMC7775213

[ref45] Khurshid Z.; Asiri F. Y. I.; Al Wadaani H. Human Saliva: Non-Invasive Fluid for Detecting Novel Coronavirus (2019-NCoV). Int. J. Environ. Res. Public Health 2020, 17, 2225 10.3390/ijerph17072225. PMC7178089 32224986

[ref46] Elberse K.; van Mens S.; Cremers A. J.; Meijvis S. C. A.; Vlaminckx B.; de Jonge M. I.; Meis J. F.; Blauwendraat C.; van de Pol I.; Schouls L. M. Detection and Serotyping of Pneumococci in Community Acquired Pneumonia Patients without Culture Using Blood and Urine Samples. BMC Infect. Dis. 2015, 15, 1–10. 10.1186/s12879-015-0788-0. 25885896 PMC4330648

[ref47] Cima-Cabal M. D.; Méndez F. J.; Vázquez F.; Aranaz C.; Rodríguez-Álvarez J.; García-García J. M.; Fleites A.; Martínez González-Río J. M.; Molinos L.; de Miguel D.; de los Toyos J. R. Immunodetection of Pneumolysin in Human Urine by ELISA. J. Microbiol. Methods 2003, 54, 47–55. 10.1016/S0167-7012(03)00004-6. 12732421

[ref48] Cima-Cabal M. D.; Vázquez F.; de los Toyos J. R.; Méndez F. J. Rapid and Reliable Identification of Streptococcus Pneumoniae Isolates by Pneumolysin-Mediated Agglutination. J. Clin. Microbiol. 1999, 37, 1964–1966. 10.1128/jcm.37.6.1964-1966.1999. 10325355 PMC84997

[ref49] Wang Y.; Wang Y.; Li D.; Xu J.; Ye C. Detection of nucleic acids and elimination of carryover contamination by using loop-mediated isothermal amplification and antarctic thermal sensitive uracil-DNA-glycosylase in a lateral flow biosensor: application to the detection of Streptococcus pneumoniae. Microchim. Acta 2018, 185, 212 10.1007/s00604-018-2723-8. 29594577

[ref50] Barbosa J. INDICATORS | Acid-Base. In Encyclopedia of Analytical Science - Reference Module in Chemistry, Molecular Sciences and Chemical Engineering; Worsfold P., Townshend A., Poole C., Eds.; Elsevier, 2005, pp 360–371 10.1016/B0-12-369397-7/00270-3.

[ref51] Gonzalez C.; Touraud E.; Spinelli S.; Thomas O. Organic Constituents. In UV-Visible Spectrophotometry of Water and Wastewater; Thomas O., Burgess C., Eds.; Elsevier, 2007; pp 47–87.

[ref52] González-González E.; Lara-Mayorga I. M.; Rodríguez-Sánchez I. P.; Zhang Y. S.; Martínez-Chapa S. O.; Santiago G. T. De.; Alvarez M. M. Colorimetric Loop-Mediated Isothermal Amplification (LAMP) for Cost-Effective and Quantitative Detection of SARS-CoV-2: The Change in Color in LAMP-Based Assays Quantitatively Correlates with Viral Copy Number. Anal. Methods 2021, 13, 169–178. 10.1039/d0ay01658f. 33399137

[ref53] Swain G. M. Solid Electrode Materials: Pretreatment and Activation. In Handbook of Electrochemistry; Zoski C. G., Ed.; Elsevier, 2007; pp 111–153.

[ref54] Yang G.; Qu X.; Shen M.; Wang C.; Qu Q.; Hu X. Electrochemical Behavior of Lead(II) at Poly(Phenol Red) Modified Glassy Carbon Electrode, and Its Trace Determination by Differential Pulse Anodic Stripping Voltammetry. Microchim. Acta 2008, 160, 275–281. 10.1007/s00604-007-0881-1.

[ref55] Schneider C. A.; Rasband W. S.; Eliceiri K. W. NIH Image to ImageJ: 25 Years of Image Analysis. Nat. Methods 2012, 9, 671–675. 10.1038/nmeth.2089. 22930834 PMC5554542

[ref56] Davidson J. L.; Wang J.; Maruthamuthu M. K.; Dextre A.; Pascual-Garrigos A.; Mohan S.; Putikam S. V. S.; Osman F. O. I.; McChesney D.; Seville J.; Verma M. S. A Paper-Based Colorimetric Molecular Test for SARS-CoV-2 in Saliva. Biosens. Bioelectron.: X 2021, 9, 100076 10.1016/j.biosx.2021.100076. 34423284 PMC8364207

[ref57] Xia Y.; Guo X. G.; Zhou S. Rapid detection of Streptococcus pneumoniae by real-time fluorescence loop-mediated isothermal amplification. J. Thorac. Dis. 2014, 6, 1193–1199. 10.3978/j.issn.2072-1439.2014.07.29. 25276360 PMC4178110

