# Supplementary material for: Electrochemical Detection for Isothermal Loop-Mediated Amplification of Pneumolysin Gene of Streptococcus pneumoniae Based on the Oxidation of Phenol Red Indicator
Source: Anal Chem. 2022 Sep 15;94(38):13061–7. doi: 10.1021/acs.analchem.2c02127 (PMC9523611; doi:10.1021/acs.analchem.2c02127)
Supplement: Supplementary file 1 — ac2c02127_si_001.pdf [file ac2c02127_si_001.pdf]

Electrochemical detection for isothermal loop-mediated amplification of pneumolysin gene of *Streptococcus pneumoniae* based on the oxidation of phenol red indicator

Andrea González-López[‡†], María Dolores Cima-Cabal[‡§], Pablo Rioboó-Legaspi[†], Estefanía Costa-Rama[†], María del Mar García-Suárez*[§], M. Teresa Fernández-Abedul*[†]

[†]Departamento de Química Física y Analítica, Universidad de Oviedo, Avda. Julián Clavería 8, 33006, Oviedo, Spain
[§]Escuela Superior de Ingeniería y Tecnología, Universidad Internacional de La Rioja, Avda. de la Paz 137, 26006 Logroño, Spain

[‡]Both authors contributed equally to this work

Contents

1. Chemical structure of phenol red (Figure S1)
2. Visual and electrochemical phenol red behavior at different pH values (Figure S2 and Table S1)
3. Successive CVs of phenol red recorded on the same electrode (Figure S3)
4. Box and whiskers plot with the values for the PR peak potential values in LAMP reactions (Figure S4)
5. Calibration curves obtained using the intensity of the green (Image J software), (Figure S5)

1. Chemical structure of phenol red

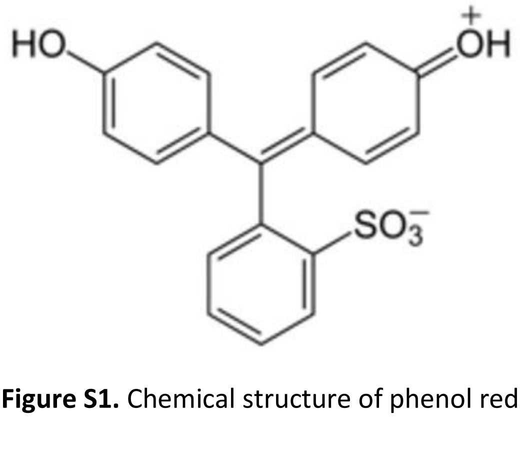

Figure S1. Chemical structure of phenol red.

2. Visual and electrochemical phenol red behavior at different pH values

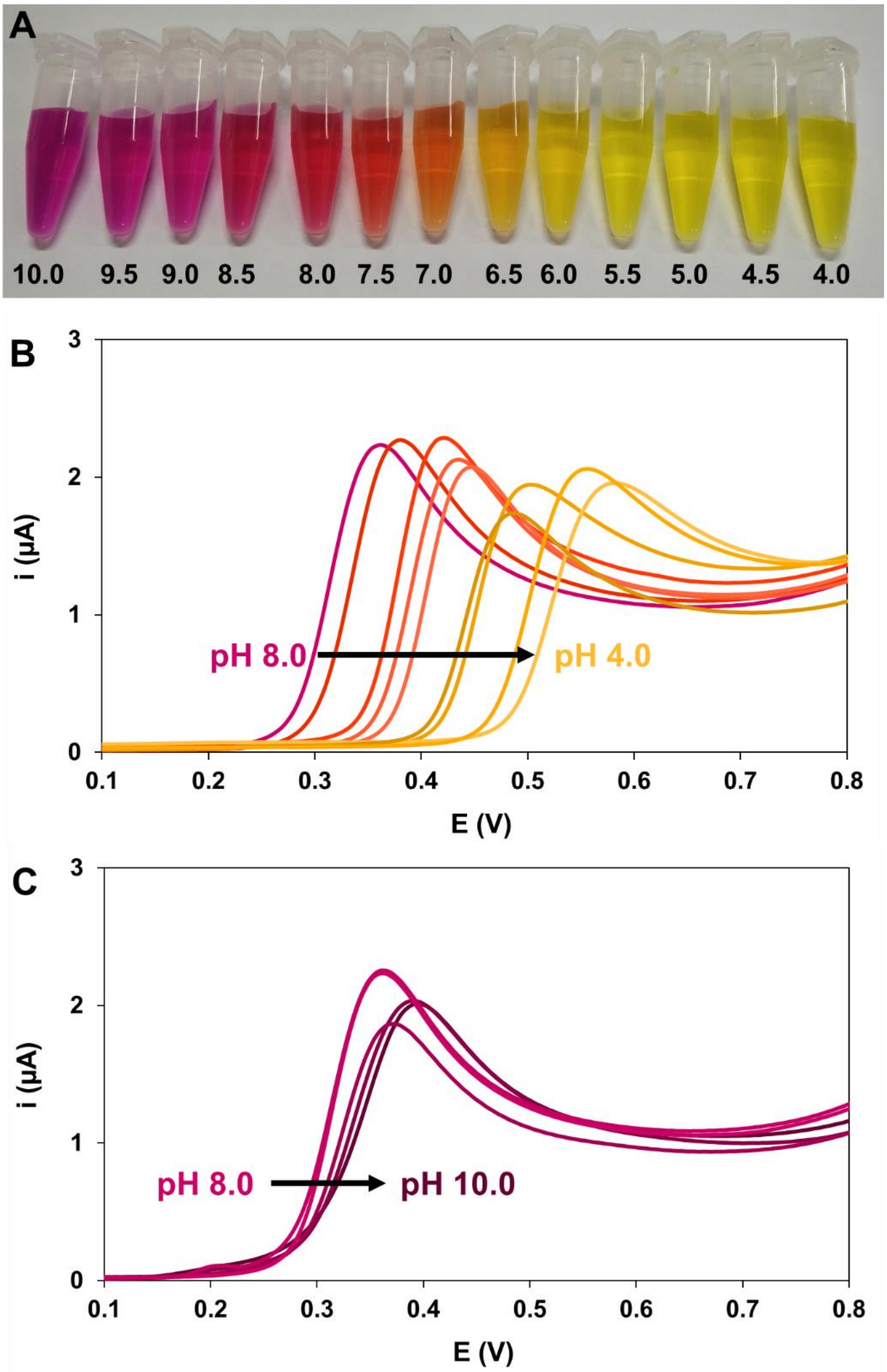

Figure S2. (A) Photograph of 0.1 mM solutions of phenol red in BR buffer solutions of different pH values. **(B)** LSVs of 0.1 mM solutions of phenol red in BR buffer solutions with pH ranging from 4.0 to 8.0 and **(C)** from 8.0 to 10.0.

Table S1. Values of peak potential and peak current intensity of voltammograms represented in Figures S2B and S2C.

pH	E_p (mV)	i_p (µA)
4.0	575	1.40
4.5	550	1.54
5.0	489	1.59
5.5	492	1.38
6.0	436	1.69
6.5	435	1.78
7.0	416	1.88
7.5	372	1.99
8.0	364	1.89
8.5	366	1.77
9.0	381	1.45
9.5	386	1.73
10.0	391	1.56

3. Successive CVs of phenol red recorded on the same electrode

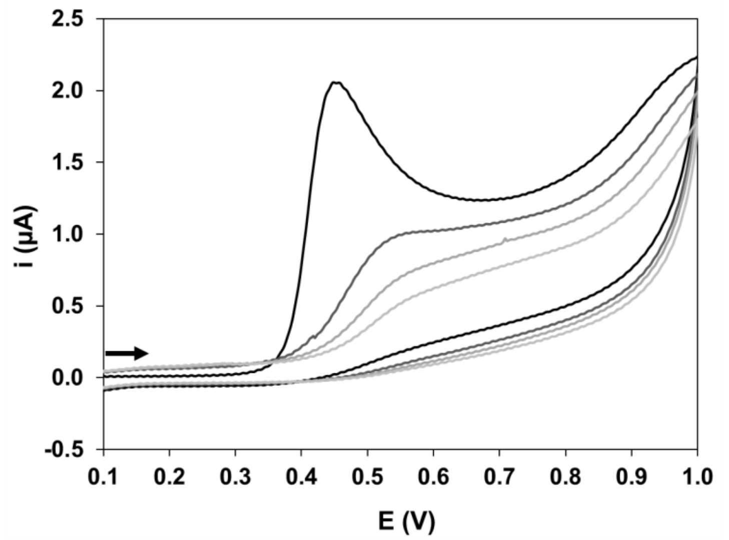

Figure S3. Four successive CVs (from black to light grey) recorded in a 0.1 mM phenol red solution in BR buffer pH 6.0, on the same electrode.

4. Box and whiskers plot with the values for the PR peak potential values in LAMP reactions

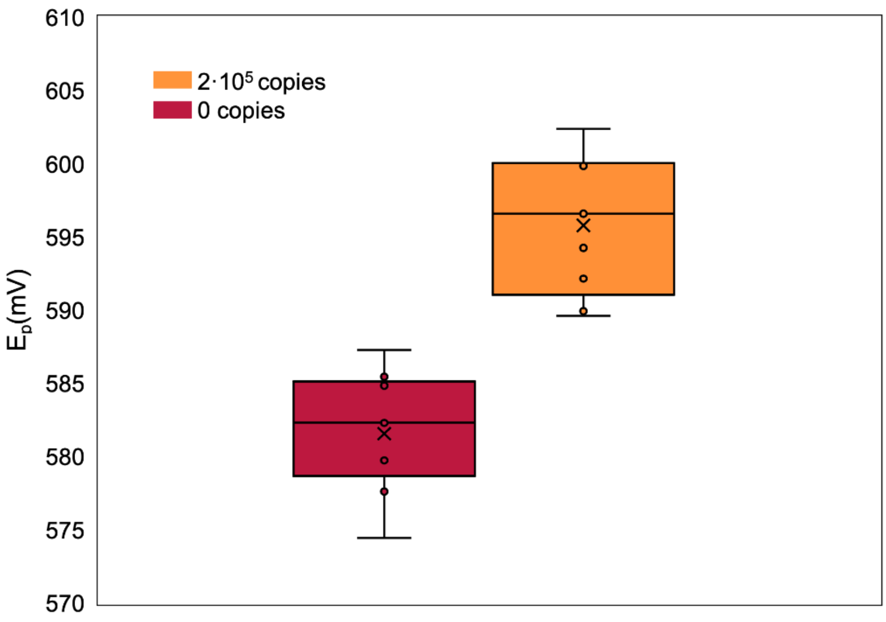

Figure S4. Box and whiskers plot showing the analytical signal for 10 negative and 10 positive (2 10^5 copies) LAMP reactions.

5. Calibration curves obtained using the intensity of the green (Image J software)

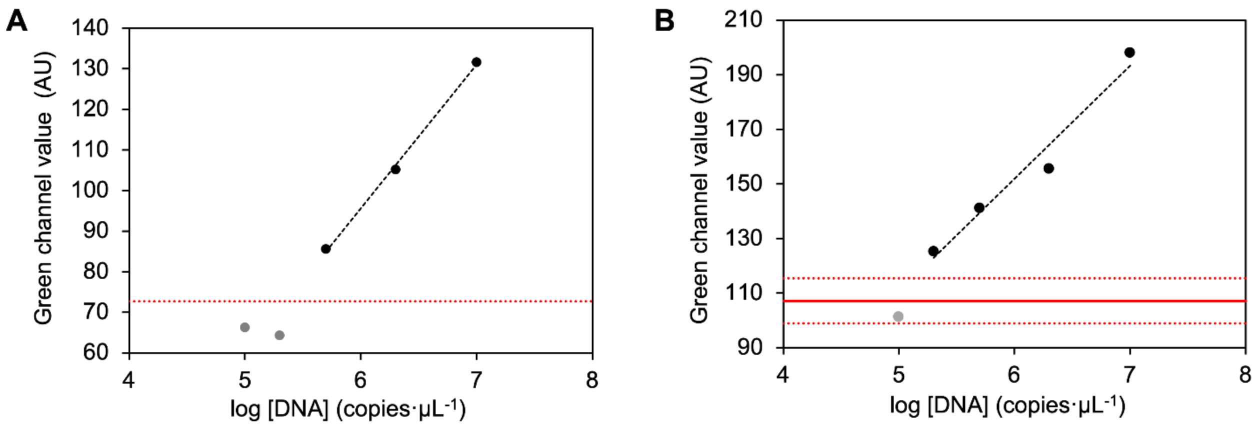

Figure S5. Calibration curves obtained representing the intensity of green from images captured for end-point LAMP reactions and treated with the open-source image processing software ImageJ for: **(A)** different initial DNA dilutions in water (the intensity of green for the negative control is represented with a red line), and **(B)** urine samples spiked with different concentrations of DNA (the mean intensity value for the negative control is represented with a continuous red line; Dashed lines represent the standard deviation of this mean).
